# Dexamethasone Inhibits the Pro-Angiogenic Potential of Primary Human Myoblasts

**DOI:** 10.3390/ijms22157986

**Published:** 2021-07-26

**Authors:** Eva K. Langendorf, Pol M. Rommens, Philipp Drees, Ulrike Ritz

**Affiliations:** Department of Orthopedics and Traumatology, University Medical Center of the Johannes Gutenberg University Mainz, 55131 Mainz, Germany; eva.langendorf@unimedizin-mainz.de (E.K.L.); pol.rommens@unimedizin-mainz.de (P.M.R.); philipp.drees@unimedizin-mainz.de (P.D.)

**Keywords:** dexamethasone, human primary myoblasts, HUVECs, co-culture, CD31, VEGF

## Abstract

Tissue regeneration depends on the complex processes of angiogenesis, inflammation and wound healing. Regarding muscle tissue, glucocorticoids (GCs) inhibit pro-inflammatory signalling and angiogenesis and lead to muscle atrophy. Our hypothesis is that the synthetic GC dexamethasone (dex) impairs angiogenesis leading to muscle atrophy or inhibited muscle regeneration. Therefore, this study aims to elucidate the effect of dexamethasone on HUVECs under different conditions in mono- and co-culture with myoblasts to evaluate growth behavior and dex impact with regard to muscle atrophy and muscle regeneration. Viability assays, qPCR, immunofluorescence as well as ELISAs were performed on HUVECs, and human primary myoblasts seeded under different culture conditions. Our results show that dex had a higher impact on the tube formation when HUVECs were maintained with VEGF. Gene expression was not influenced by dex and was independent of cells growing in a 2D or 3D matrix. In co-culture CD31 expression was suppressed after incubation with dex and gene expression analysis revealed that dex enhanced expression of myogenic transcription factors, but repressed angiogenic factors. Moreover, dex inhibited the VEGF mediated pro angiogenic effect of myoblasts and inhibited expression of angiogenic inducers in the co-culture model. This is the first study describing a co-culture of human primary myoblast and HUVECs maintained under different conditions. Our results indicate that dex affects angiogenesis via inhibition of VEGF release at least in myoblasts, which could be responsible not only for the development of muscle atrophy after dex administration, but also for inhibition of muscle regeneration after vascular damage.

## 1. Introduction

The tightly regulated process of angiogenesis and the formation of new blood vessels is a requirement for intact tissue as well as regeneration. The regulation is controlled by growth factors, chemokines, endothelial cell-specific receptors and adhesion molecules [1]. Impaired angiogenesis is associated with various pathological conditions including cancer, diabetic retinopathy, asthma, autoimmune disorders and infectious diseases [2,3]. Moreover, angiogenesis plays an important role in organ growth and wound healing [4,5], as blood vessels supply tissues with oxygen and nutrients and remove waste products [6]. Vascularization, essential in wound healing, is regulated by several growth factors: basic fibroblast growth factor (bFGF) [7], platelet derived growth factor (PDGF), epidermal growth factor (EGF) and vascular endothelial growth factor (VEGF) [8]. Pro-inflammatory mediators e.g., interleukins and interferons stimulate the endothelium to produce various chemokines [9,10,11,12]. VEGF activates endothelial cells to proliferate, migrate, and to form sprouts. Moreover, VEGF is involved in blood vessel formation, wound healing, and organ regeneration [13,14]. VEGF increases myoblast migration and inhibits apoptosis in the skeletal muscle in vivo [15,16]. Moreover, it is suggested that VEGF can modulate myoblasts, which contributes to the recruitment of progenitor cells in regenerating muscle. Therefore, it is an indicator for common processes with identical triggers for regeneration and angiogenesis [17,18].

Glucocorticoids (GCs) are endogenous stress hormones with anti-inflammatory properties and used to treat inflammatory disorders [19,20]. GCs decrease the production of several pro-inflammatory cytokines and chemokines. including IL-6 [21,22,23] and thereby limiting inflammation [24]. However, high therapeutic doses and prolonged phases of medication can induce undesired side-effects, including osteoporosis, diabetes and hypertension [25]. Additionally, GCs inhibit pro-inflammatory signalling [26] and angiogenesis. In fact, suppression of angiogenesis contributes to impaired wound healing caused by GC excess.

Previous studies suggest that GCs inhibit the process of angiogenesis by downregulation of the glucocorticoid receptor (GR) [27], resulting in the inhibition of VEGF [28,29]. Others found that GCs blocked the VEGF-induced tube-like-structure (TLS) of endothelial cells, but without any effects on proliferation or migration [15]. This resulted in a decreased formation of cell–cell contacts. Similar effects by using high and prolonged doses of GCs can result in skeletal muscle atrophy, caused by the catabolic effects [30] and further in muscle wasting [31,32]. Dex impact on primary human myoblasts was investigated in our previous study [33]. We found that dex reduced protein expression of myosin heavy chain (MyHC) and elevated the expression of the atrophy related ubiquitin ligase MuRF-1. However, beside the fact that only limited studies with human myoblasts exist, neither the mechanisms nor the interaction between myoblasts or endothelial cells under the influence of dex has been described. There are no reports describing the effects of dex on both cell types in a co-culture system in vitro, which could show and analyze the mechanisms of dex administration on the interaction of these two cell types.

The hypoxia-inducible transcription factor-1 (*HiF-1*) increases angiogenesis in normal tissues [34]. It is reported that hypoxia reduces the GR expression and attenuates the anti-inflammatory effects of GCs [35,36]. Dex impairs *HiF-1α* gene expression [37], but the GR activation is associated with enhanced HiF-1 activity, which is partly in contrast to other studies [38]. GCs play a key role in the suppression of inflammation by inhibiting the transcription of the cytokines through binding to the GR and the activated GR interacts with several transcription factors in a positive or negative manner [39].

Our hypothesis is that dexamethasone impairs angiogenesis leading to muscle atrophy or inhibited muscle regeneration. Therefore, this study aims to elucidate the effect of dexamethasone (dex) on HUVECs under different conditions in mono- and co-culture with myoblasts to evaluate growth behavior and dex impact with regard to muscle atrophy and muscle regeneration. We investigated the effect of dex on endothelial cells under different growth conditions and in various environments. Additionally, we analyzed the influence of VEGF under the previously described conditions. In a second part, we established and evaluated a co-culture system of primary human myoblasts and HUVECs to analyze their growth behavior and interaction after dex administration in order to define effects leading to muscle atrophy and inhibited muscle regeneration.

## 2. Results

### 2.1. Dex Influences Cell Viability Depending on the Presence of VEGF

**Figure 1:**
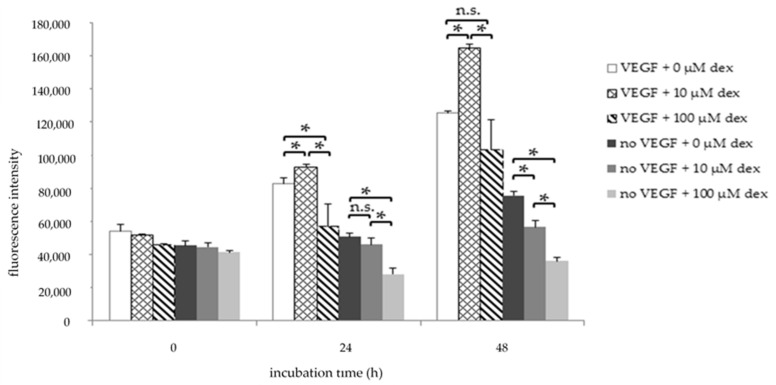
Viability assay of HUVECs after 0, 24, and 48 h in HM1 medium (with VEGF) or in HM1-V medium (without VEGF) and dex (10, 100 µM). Results are presented as bar charts; error bars show the standard deviation (*n* = 3). A *p* value < 0.05 indicate statistical significance (* *p* < 0.05), n.s. = non-significant.

To investigate the influence of dex on HUVEC viability and proliferation, the AlamarBlue^®^ cell viability assay was performed after 24 h and 48 h treatment. HUVECs were treated with 10 µM or 100 µM Dex, or left untreated (control), in medium containing VEGF (HM1) or without VEGF (HM1-V) (Figure 1). The treatment of HUVECs with 10 µM dex resulted in a significantly increased fluorescence intensity corresponding to better viability and faster proliferation compared to the untreated control after 24 and 48 h in HM1 (Figure 1). In contrast, proliferation of cells treated with 100 µM was significantly lower compared to the control group and to the group with 10 µM dex in HM1 after the same time points.

When VEGF was excluded from the medium (HM1-V), 10 µM and 100 µM Dex treatment decreased HUVEC cell viability most pronounced at 48 h, but also already at 24 h, compared to the control (Figure 1).

In order to show that the effect was specific to dex we performed the same experiment with the GR-antagonist RU-486. The inhibitor reversed the dex effects, demonstrating the specificity (Figure S1).

### 2.2. Dex Inhibits TLS Formation Depending on VEGF Presence 

**Figure 2:**
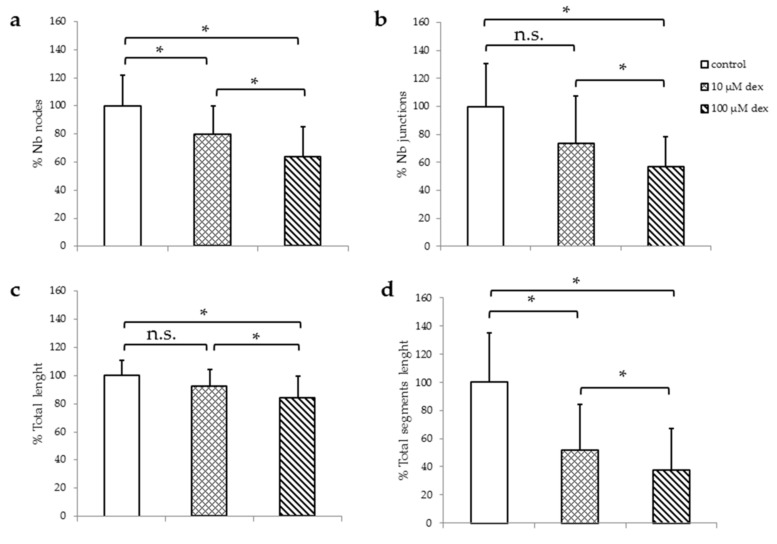
TLS assay of HUVECs after 24 h in HM1 with 10 and 100 µM dex. Results are presented as bar charts; error bars show the standard deviation (*n* = 3). Quantitative analyzes were performed for number of nodes (**a**), number of junctions (**b**), total length (**c**), and the total segments length (**d**). Data were normalized to a control of 100%. A *p* value < 0.05 indicate statistical significance (* *p* < 0.05), n.s. = non-significant.

In order to analyze HUVECs ability to form their typical TLS, they were seeded in Matrigel in HM1 (Figure 2) or in HM1-V medium (Figure S2) and incubated with or without dex for 24 h. The percentage of the number of nodes, junctions, total length, and the total segment length was analyzed and compared to the untreated control. Representative example images of the TLS assay are shown in the Supplementary Materials (Figure S2).

The number of nodes as well as the total segment length were significantly reduced after the incubation with 10 µM (Figure 2a). Incubation with 100 µM resulted in a significant decrease in all parameters measured.

Investigations of HUVECs in HM1-V medium revealed no significant changes in the number of nodes, junctions, total length, and total segmental lengths 24 h after incubation with 10 µM dex compared to untreated control. However, incubation with 100 µM resulted in a significant decrease in all parameters measured (Figure S3).

In summary, the higher the amount of dex, the higher the reduction of the number of nodes, number of junctions, total length and total segment length. Moreover, in the presence of VEGF, dex reduces TLS formation even further.

### 2.3. The Influence of Dex on Gene Expression Is Independent of the Cells Growing in a 2D or 3D Matrix

**Figure 3:**
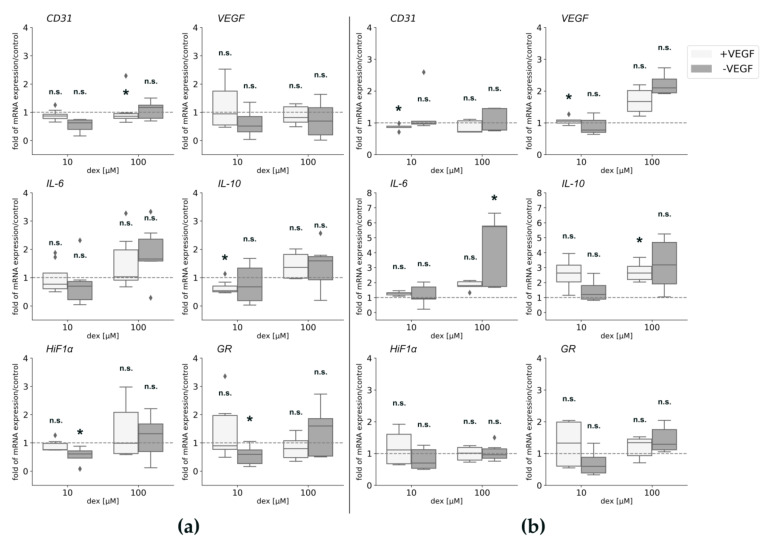
Gene expression analyses for *CD31*, *VEGF*, *IL-6*, *IL-10*, *HiF1α*, and *GR* of HUVECs in HM1 medium (with VEGF) or in HM1-V medium (without VEGF) and 10 and 100 µM dex for 24 h. HUVECs were seeded in Matrigel (**a**) or on plastic culture plates (**b**). The mRNA levels were normalized to *GAPDH* and calculated as ratios in relation to the untreated control group (interrupted line). Results are presented as medians and quartiles (*n* = 3), and *p* values < 0.05 indicate statistical significance (* *p* < 0.05), n.s. = non-significant. Outliners are presented as rhombs.

To further analyze possible effects of dex under the influence of VEGF, gene expression analyses were performed for *CD31*, *VEGF*, *HiF1α*, and *GR*. In order to detect any influences of dex on inflammation, gene expression of pro-and anti-inflammatory genes *IL-6* and *IL-10* was analyzed. In order to determine possible changes in gene expression, depending on the environment, HUVECs were seeded in Matrigel (Figure 3a) or in normal plastic culture plates (Figure 3b) under the same conditions.

Regarding *CD31* gene expression changes, it was only significantly suppressed after culture in Matrigel and incubation with 100 µM dex with VEGF compared to the untreated control group. When cells were cultured on plastic gene expression of the surface protein *CD31* (Figure 3b), there was a statistically significant decrease after the treatment with 10 µM dex when cells were cultured in HM1 compared to the untreated control cells. The incubation with 100 µM dex did not influence *CD31* gene expression.

There were no statistical differences detected in *VEGF* gene expression in Matrigel compared to control cells at any condition tested (Figure 3a). When cells were cultured on plastic, only incubation with 10 µM dex led to a statistically significant upregulation in *VEGF* gene expression in VEGF containing medium. 100 µM dex led to an upregulation that was not statistically significant, however.

Analysis of the transcription factor *HiF1α* gene expression after culture in Matrigel revealed only a significant downregulation after the incubation with 10 µM dex in HM1-V. The expression of *HiF1α* (Figure 3b) after culture on cell culture plates was not regulated in any condition tested.

Regarding gene expression of pro-and anti-inflammatory genes *IL-6* and *IL-10*, no statistically significant changes were observed after culture in Matrigel with the exception that IL-10 was significantly downregulated after the treatment with 10 µM dex in HM1. Regarding culture in cell culture plates, 100 µM dex led to a significant upregulation of *IL-6* (without VEGF) and *IL-10* gene expression (with VEGF) compared to untreated control cells (Figure 3b).

The gene expression of *GR* was statistically significantly reduced when cells were cultured in Matrigel without VEGF and 10 µM. Cell culture in cell culture plates resulted in no significant gene expression changes independent from culture conditions used.

### 2.4. Cell Viability Depends on Exogenous Growth Factors

**Figure 4:**
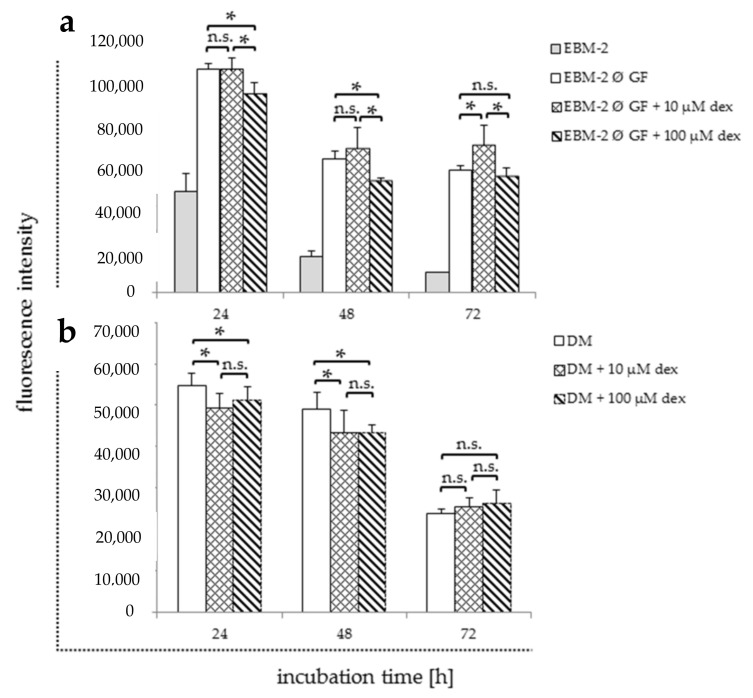
Viability assay of HUVECs 24, 48, and 72 h after the incubation with and without dex in different media compositions ((**a**): EBM-2 without growth factors; (**b**): DM). Results are presented in bar charts; error bars show the standard deviation (*n* = 3). A *p* value < 0.05 indicate statistical significance (* *p* < 0.05), n.s. = non-significant.

**Figure 5:**
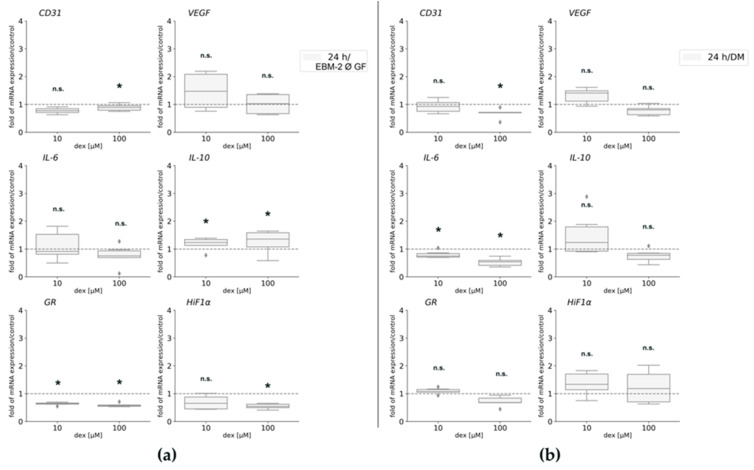
Gene expression analyses of HUVEC maintained in EBM-2 Ø GF (**a**) and in DM (**b**) for *CD31*, *VEGF*, *IL-6*, *IL-10*, *GR*, and *HiF1α*. The mRNA levels were normalized to *GAPDH* and calculated as ratios in relation to the untreated control group (interrupted line). Results are presented as medians and quartiles (*n* = 3), and *p* values < 0.05 indicate statistical significance (* *p* < 0.05), n.s. = non-significant. Outliners are presented as rhombs.

**Figure 6:**
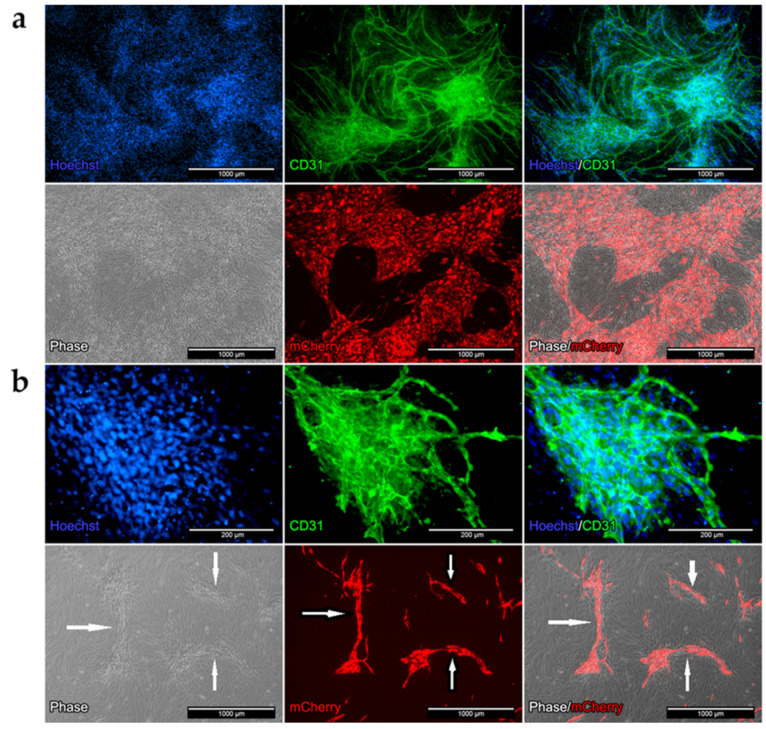
Immunofluorescence of the co-culture after 5 days in HM1/DM (**a**) and in DM (**b**) medium. Cells were stained with CD31 specific antibody (green). Nucleus staining was performed with Hoechst dye (blue). Phase contrast images of the co-culture ((**a**,**b**), grey). HUVECs transduced with the mCherry vector ((**a**,**b**), red) in the co-culture; overlay of the phase contrast images and the transduced HUVECs (red, grey). Myoblasts were not transduced.

In order to define the effect of different media and their supplements in combination with dex on HUVECs, they were analyzed after dex treatment without access to growth factors. Viability (Figure 4) and gene expression analyses (Figure 5 and Figure 6) were performed using the following media: (i) EBM-2 medium alone (EBM-2) (Table 1, negative control); (ii) EBM-2 medium with serum, hydrocortisone, heparin, and ascorbic acid, but without growth factors (EBM-2 Ø GF) and hydrocortisone either with or without dex. Additionally, cells were maintained in DM (Table 1), which was normally used for myoblast differentiation with and without dex for further co-culture experiments (Figure 4b and Figure 6).

The negative control showed a rapid reduction in cell viability over all time points (Figure 4a). The treatment with 10 µM significantly increased proliferation only after 72 h compared to the untreated EBM-2 without GF control (Figure 4a). The treatment with 100 µM significantly reduced proliferation rate compared to control after 24 and 48 h.

Maintaining cells in DM and treating them with 10 and 100 µM, dex significantly decreased the proliferation after 24 and 48 h compared to the untreated DM control (Figure 4b). However, no statistically significant differences were observed between all groups after 72 h.

### 2.5. Gene Expression Is Affected by Dex and Depends on the Media

To further analyze possible effects of dex under the influence of growth factors and different media compositions, gene expression analyses were performed for *CD31*, *VEGF*, *HiF1α*, and *GR*. In order to detect any influences of dex on inflammation, gene expression of pro-and anti-inflammatory genes *IL-6* and *IL-10* was analyzed.

*CD31* gene expression was not statistically significantly affected by 10 μM dex maintaining cells in EBM-2 Ø GF or DM (Figure 5a; Figure 5b). However, the treatment with 100 μM significantly decreased its expression compared to the untreated control cells in both media (Figure 5a,b). *VEGF* gene expression was not statistically changed after dex treatment in both media (Figure 5b). *HiF1α* gene expression was not affected by the low concentration (10 µM) but its expression was significantly downregulated after incubation with the high dex concentration (Figure 5a) in EBM-2, whereas no changes were observed after incubation in DM (Figure 5b).

Regarding gene expression of pro-and anti-inflammatory genes *IL-6* and *IL-10*, a contrary gene expression was observed: in EBM-2 media, dex had no significant effect on *IL-6* gene expression (Figure 5a), whereas gene expression of *IL-10* was statistically significantly upregulated after the treatment with 10 and 100 µM dex compared to the untreated control (Figure 5a). Concerning incubation in DM, gene expression of *IL-6* was significantly downregulated by 10 and 100 µM dex compared to the untreated control cells (Figure 5b), whereas *IL-10* gene expression changed to be not statistically significant after the treatment with both concentrations for 24 h (Figure 5b).

The gene expression of the *GR* was significantly decreased in HUVECs after dex incubation compared to the untreated cells in EBM-2 (Figure 5a). whereas no changes were observed after incubation in DM.

### 2.6. Designing a Co-Culture System of Human Endothelial Cells with Primary Myoblasts

A co-culture of primary human myoblasts and human endothelial cells has been established to assess interactions of cell types with regard to the development of muscle atrophy, muscle regeneration, and the role of angiogenesis.

First, we investigated the behavior of both cell types, especially of HUVECs, in different media compositions (Table 2) as HUVECs require a mixture of different growth factors to proliferate and survive. Figure S4 shows both cell types as mono-and co-cultures in the applied different media.

In contrast to HUVEC mono-cultures in DM (contains no growth factors, Figure S4b), HUVECs did not undergo apoptosis in co-culture with myoblasts in DM medium (Figure S4e). To explain this effect, we hypothesized that primary myoblasts produced growth factors, which allowed HUVECs to survive.

### 2.7. Formation of Vessel-Like Structures in the Co-Culture

To further define the characteristics of the co-culture, immunofluorescence staining using a specific antibody for the angiogenic marker CD31 (Figure 6) was performed. In addition, HUVECs were transduced with the mCherry-protein to distinguish the cell types.

Cells in the co-culture stained positive for CD31 protein in the medium mixture HM1/DM. In the phase contrast images, the large areas can be clearly identified as endothelial cells (Figure 6a). The CD31 staining showed wide branched, elongated, and tubular structures, but hardly any bulges. In the co-culture without growth factors (DM), cells were positive for the angiogenic marker CD31 after 5 days. In the phase contrast images, small, elongated bulges of HUVEC cells were visible (Figure 6b—white arrows). Staining with CD31 confirmed small, elongated tubes and tubular branches indicating the process of angiogenesis performed by endothelial cells.

### 2.8. Dex Reduces the Angiogenic Potiential of HUVECs in the Co-Culture

To analyze the influence of dex on bulge formation in a co-culture system, HUVECs and myoblasts were incubated for 96 h in DM and analyzed after 48 and 96 h (Figure S5).

After 48 h there were no visible differences between the untreated control and the treated groups with 10 and 100 µM dex (Figure S5). In the control as well as in both treated groups, cells started to form multiple bulges. After 96 h (Figure S5) fewer and less pronounced bulges were visible in the treated groups with 10 and 100 µM dex compared to the untreated control group. Moreover, the bulges in the control seemed to be stronger than in the treated groups.

### 2.9. Dex Impairs Angiogenesis and Decreases CD31 Protein Expression in the Co-Culture

**Figure 7:**
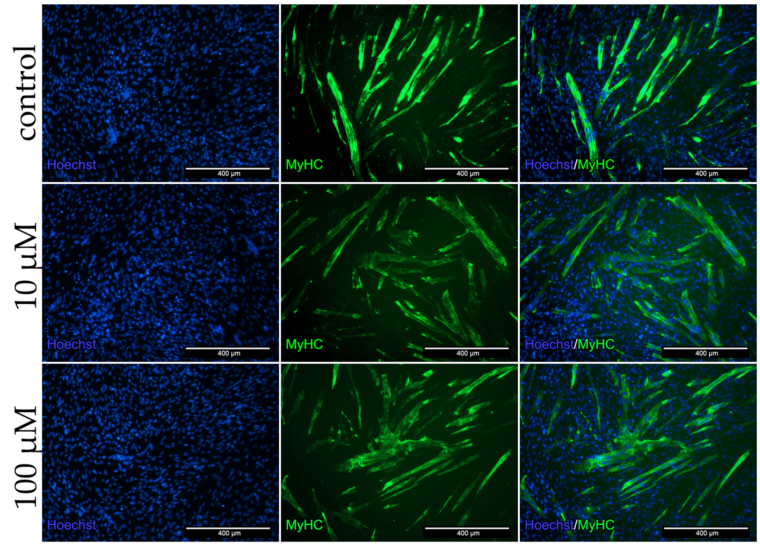
Immunofluorescence staining for MyHC after 96 h incubation in DM with 10 and 100 µM dex in the co-culture and untreated control. Myotubes were stained with MyHC (MyHC, green) specific antibody. Nucleus staining was performed using Hoechst dye (blue).

**Figure 8:**
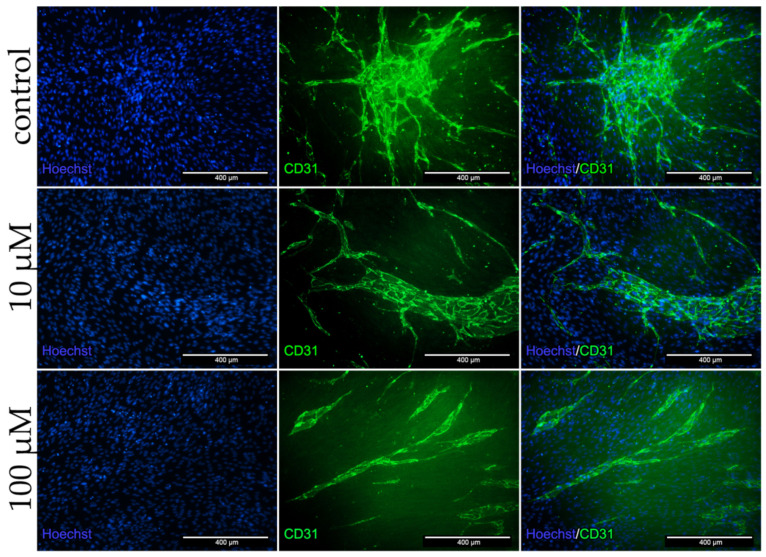
Immunofluorescence staining for CD31 after 96 h incubation in DM with 10 and 100 µM dex and untreated control in the co-culture. HUVECs were stained with CD31 (green) specific antibody. Nucleus staining was performed using Hoechst dye (blue).

To analyze the protein expression in the co-culture as well as the influence of dex on cells, stainings for the myotube marker MyHC (Figure 7) and the angiogenic marker CD31 (Figure 8) were performed.

Positive stained cells for the myotube marker MyHC and the formation of multinucleated cells were observed in all groups (Figure 7). The qualitative analysis showed no reduction in the formation of multinucleated myotubes and myotube diameter after dex treatment.

Figure 8 shows the positive CD31 antibody staining of HUVECs after 96 h in the co-culture. In the control group a close meshed formation of TLS could be detected. Both dex treated group showed no close meshed bulges, but rather long sustained and undefined structures (Figure 8).

To summarize, CD31 and MyHC staining of co-cultures revealed that dex had a higher impact on endothelial cells than on human myoblasts.

### 2.10. Dex Reduces VEGF Secretion of Myoblast and Decreases Angiogeneses

**Figure 9:**
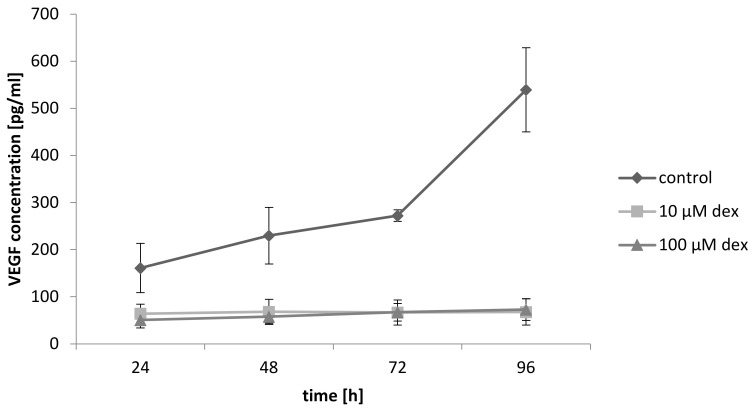
VEGF release in the co-culture with and without dex after 24, 48, 72, and 96 h. Results are represented in a line diagram and error bars show the standard deviation (*n* = 3).

To determine if primary myoblasts produce VEGF and whether this secretion was influenced after dex treatment, a VEGF ELISA was performed. The results presented in Figure 9 shows that VEGF release increased continuously in the co-culture in the control group. Treatment with 10 and 100 µM dex drastically reduced the released amount of VEGF. No differences in the released amount of VEGF between 10 and 100 µM dex were observed.

### 2.11. Dex Enhances the Expression of Myogenic Transcription Factors but Represses Angiogenesis in the Co-Culture

**Figure 10:**
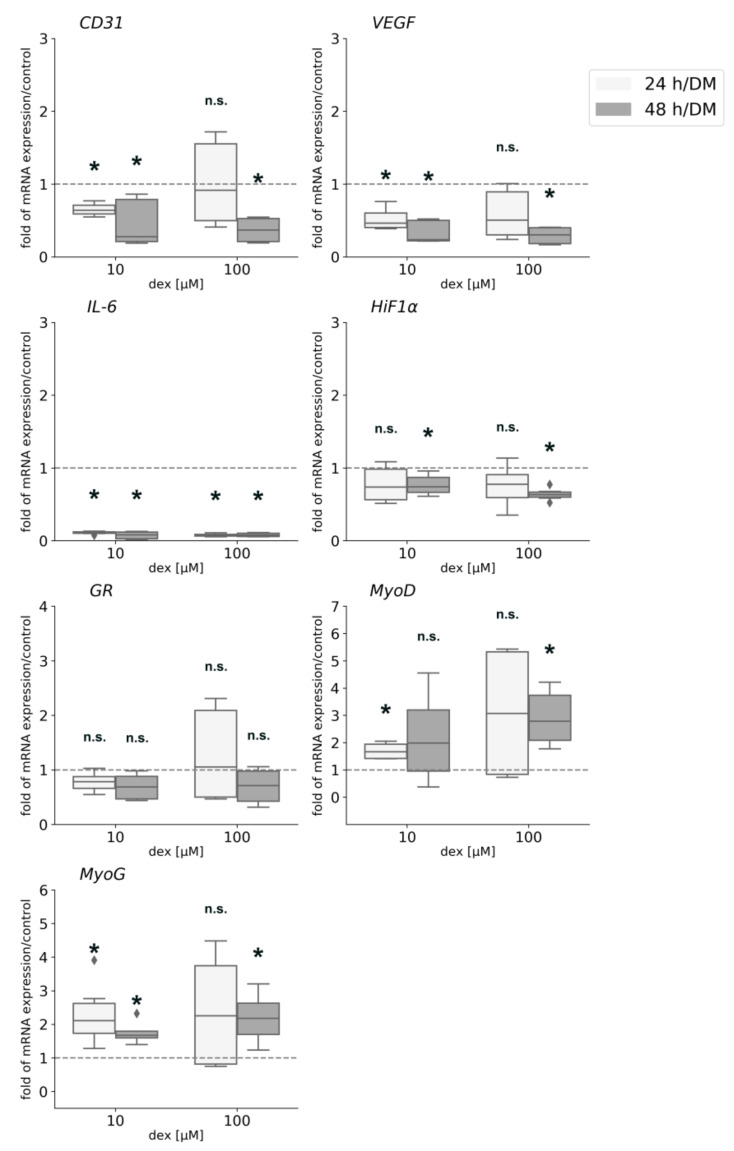
Gene expression analyses for *CD31*, *VEGF*, *IL-6*, *HiF1α*, *GR*, *MyoD*, and *MyoG* in the co-culture after 24 and 48 h in DM with or without 10 or 100 µM dex. The mRNA levels were normalized to *GAPDH* and calculated as ratios in relation to the untreated control group (interrupted line). Results are presented as medians and quartiles (*n* = 3), and *p* values < 0.05 indicate statistical significance (* *p* < 0.05), n.s. = non-significant. Outliners are presented as rhombs.

In order to determine the effects of dex on gene expression in the co-culture system, qPCR analyses for various markers were performed (Figure 10).

*CD31* and *VEGF* gene expression was significantly downregulated when cells were incubated with 10 µM dex for 24 h and 48 h and when they were incubated with 100 µM for 48 hours. Gene expression of *IL-6* was significantly downregulated after both incubation times and both concentrations of dex. *IL-10* gene expression was not influenced by dex treatment (data not shown). Gene expression analysis of *HiF1α* was only significantly downregulated 48 h after the incubation with 10 and 100 µM. The expression of the *GR* was not influenced by dex, neither after 24 nor after 48 h. The myogenic factor *MyoD* showed a significantly higher expression level after 24 h incubation with 10 µM dex and after 48 h with 100 µM dex in the co-culture. The transcription factor *MyoG* was significantly upregulated by 10 µM after 24 and 48 h, but 100 µM influenced and upregulated its expression only after 48 h.

For our studies, we used dex concentrations of 10 and 100 µM. Regarding studies with glucocorticoids, various concentrations are used ranging from 10 nM to 100 µM. We decided to use these two concentrations as Han et al. [40] demonstrated that 100 µM dex exhibited the most prominent atrophic effect on C2C12 myoblasts. Moreover, Shin et al. showed that C2C12 myotubes responded in a dose-dependent manner to increasing dex concentrations (10–50–100 µM) [41]. Wang et al. [42] and others [43] observed that 100 µM dex suppressed muscle protein synthesis and enhanced proteolysis—thereby representing a potential model of muscle atrophy or inhibited muscle regeneration [44]. Doses in the nanomolar range stimulate myogenesis, and thus why we excluded these concentrations from our setting. Moreover, we and others [33] could show that the applied high concentrations at least partially induce myogenic gene expression. As we wanted to imitate atrophic conditions as described in the cited literature, we chose the concentrations of 10 and 100 µM to characterize the effects on HUVEC, which has, to our knowledge, not been analyzed before.

## 3. Discussion

### 3.1. Dex Influence on Endothelial Cells Cultured in Mono-Culture Depends on Different Factors

Our results show that 10 µM dex significantly enhanced cell proliferation, whereas 100 µM caused the opposite effect (Figure 1). It has already been demonstrated that high concentrations of GCs inhibit the proliferation of human endothelial cells [45]. The increase in proliferation after cells were treated with 10 µM dex could result from the hydrocortisone supplementation in the medium as well as from an additional low concentration of dex leading to an increase in cellular stress. Cells cultured in medium without VEGF had a slower proliferation rate and dex had a higher effect on HUVECs when VEGF was absent. These results confirm that VEGF plays a central role in the proliferation of HUVECs [15]. Our results show that high dex concentrations repress endothelial cell viability and proliferation and confirm its anti-angiogenic effect. This effect was reversed after incubation with the GR antagonist RU-486, showing the specific effect of dex.

To figure out the influence of other growth factors in combination with dex on HUVECs viability and proliferation, cells were maintained in different media with different supplements and with and without dex. Our results showed that HUVECs require at least a small portion of supplements to survive, but they have the ability to readapt to reduced growth conditions within 24 h. Similar results were obtained in a study using cortisol [15]. We also observed a lower influence of dex in the presence of all growth factors, especially VEGF, indicating that growth factors play a major role on how dex can affect endothelial cells. This hypothesis was confirmed by the results obtained when HUVECs were grown in DM. Regarding TLS formation, we could show that the higher the concentration of dex, the more influenced each single component was e.g., building of nodes or junctions. The results in the present study clarify the immediate relation between the exogenous dex and its ability to inhibit the VEGF-induced TLS formation in endothelial cells, which has also been reported in another study [46]. This inhibition could be explained by the fact that dex decreases cell–cell contacts [15] and influences cell proliferation, which was also confirmed in our study (Figure S2). The dex-induced inhibition was VEGF-mediated at low concentrations, whereas higher concentrations seemed to be VEGF independent.

In conclusion, our results show that dex effects on cell viability and TLS formation of HUVECs depend on growth factors, especially on VEGF.

Gene expression analyses of CD31 on HUVECs maintained in mono-culture in different media confirmed that higher concentrations of dex have a high impact on angiogenesis and that growth factors play an important role in the process of angiogenesis. The statistically significant downregulation of *CD31* gene expression after 100 µM dex treatment for 24 h in Matrigel in HM1 medium indicated that the high concentration reduced the angiogenesis process in HUVECs. Interestingly, the exclusion of VEGF (HM1-V) led to no statistically significant downregulation of *CD31* gene expression suggesting that VEGF was not in itself an important factor for the induction of angiogenesis.

*VEGF* gene expression was not or only minimally influenced at any condition tested (Figure 3a). These results were unexpected, as it was reported that GCs inhibit the process of angiogenesis by inhibiting *VEGF* [28,29,47,48]. Nagashima et al. found that dex inhibited *VEGF* gene expression as a result of dex treatment [49]. Only one other study showed that dex did not influence *VEGF* gene expression of endothelial progenitor cells [50], confirming the findings in the present study with HUVECs.

Gene expression of the pro-inflammatory cytokine *IL-6* was influenced in some conditions tested. Only 100 µM dex induced a significant upregulation of *IL-6* gene expression (Figure 3b) compared to the untreated control when cells were seeded on normal plastic culture plates without VEGF in the medium (Figure 3b). Downregulation by dex was observed in HUVECs cultured in DM after 10 and 100 µM dex treatment compared to the untreated control, which was also reported in previously published studies [21,22,23]. Normally, *IL-6* gene is only activated during inflammation and dex decreases the production of IL-6 cytokines [21,22,23]. As we did not induce inflammatory conditions in this study, we did not expect an *IL-6* upregulation under these normal conditions.

Gene expression of the anti-inflammatory cytokine *IL-10* was either not regulated (DM-medium) or upregulated after 10 and 100 µM dex compared to control (EBM-2 without GF). These results indicated that dex had the same impact on *IL-10* expression in HUVECs as in monocytes [51]. Gene expression of the anti-inflammatory cytokine *IL-10* was (except 10 µM dex in HM1 medium) not statistically significantly downregulated compared to the control and independent of VEGF (Figure 3a). *IL-10* inhibits the production of pro-inflammatory cytokines, such as *IL-6* reducing the inflammation process under inflammatory conditions [52]. It has been demonstrated that dex increases the gene expression of *IL*-*10* in monocytes under normal conditions [51]. However, we detected neither a significant downregulation nor significant expression changes of *IL-10* under normal conditions or when VEGF was not in the medium. This indicates that this cytokine is not influenced in endothelial cells when treated with dex under normal conditions.

The significant downregulation of the *GR* after 10 as well as after 100 µM dex addition, when cultured in EBM-2 without GF or in medium without VEGF, was also observed in our pervious study in primary human myotubes [33].

The concentration of 10 µM did not alter *HiF1α* expression, whereas 100 µM significantly downregulated its expression (Figure 5a), indicating that high concentrations lead to the inhibition of *HiF1α* resulting in the reduced expression of the *GR*, which has already been described in another study [36]. It also indicates that dex impairs the function of HiF1α, described by Elsby et al. [53], although others found that dex induced the repression of *HiF1α* expression [37]. The gene expression of *HiF1α* was only significantly downregulated after maintaining in HM1-V medium and the incubation with 10 µM compared to the untreated control cells. These results suggest that the influence of dex on *HiF1α* gene expression was independent of VEGF under non hypoxic conditions. Other studies reported that dex induced the suppression of *HiF1α* [37] and that dex suppressed the expression of the *HiF1* target gene VEGF in HepG2 cells [36]. However, our results show that dex did not influence *HiF1α* under normal conditions and *HiF1α* remained continuously expressed, demonstrating that dex did not repress *HiF1α* in endothelial cells under dex induced conditions and that this effect was independent of VEGF (Figure 3a).

Our findings indicate that the expression of *HiF1α* depends on the GR. Similar findings were observed in HeLa cells [38]. They found that *HiF1α* -dependent gene expression is upregulated by GCs via the GR and that its activation is associated with *HiF1α* upregulation.

In conclusion, the gene expression analyses confirm the findings that regulation by dex depends on the growth factor and cytokine environment of the cultured cells, whereas culture conditions regarding 2D or 3D play a minor role.

### 3.2. Effects of Dex in a Co-Culture System of Human Endothelial Cells with Primary Myoblasts

#### 3.2.1. Formation of Vessel-Like Structures in the Co-Culture

To our knowledge, no co-culture models consisting of human endothelial cells and human primary myoblasts to analyze cell–cell interactions in regard to muscle regeneration or development of muscle atrophy exist. Therefore, we established a co-culture model of these cell types in different cell media and characterized it regarding angiogenesis, gene expression and proliferation. We observed a strong angiogenesis in the co-cultures independent from the media applied—even without applied growth factor, in particular, without VEGF (Figure 6). Our hypothesis is that this effect has been caused by a VEGF release from human primary myoblasts comparable to the release of VEGF described in murine myoblasts [54,55]. Our results indicate that by VEGF release of human myoblasts, the vascular development is regulated and induced in our co-culture system.

In co-culture, a clear and directed cell formation of HUVECs could be observed (Figure 6), which suggested the migration and differentiation of HUVECs due to the VEGF release by myoblasts (Figure 9). A similar cell formation of a capillary network has been described in murine myoblasts [56,57].

These results indicate an enhanced angiogenesis through maintaining HUVECs and myoblasts together even without additional growth factors, indicating a positive influence of myoblasts on HUVECs and angiogenesis.

To analyze the effect of dex on cell behavior and angiogenesis, the co-culture was incubated with 10 and 100 µM dex for 48 and 96 h. The results show that already low concentrations of dex affected the formation of bulges in HUVECs, including cell migration clearly indicating that dex had a higher influence on HUVECs than on the other cell types. The observations suggest that dex inhibited the process of angiogenesis similar to the process previously described for keratinocytes [28,29]. In the mentioned studies, the authors suggested that angiogenesis is impaired by dex inhibiting VEGF expression. This might be an explanation for the findings of the experiments using DM medium without growth factors in combination with dex. Although the medium contains no growth factors, HUVECs proliferated as VEGF is released by myoblasts. Our hypothesis is that dex caused a reduction of the VEGF-release, which resulted in a reduced microvessel density. Similar results have been obtained in a study with prostate cancer cells [27] and in a mono-culture of myoblasts [58].

#### 3.2.2. Dex Impairs Angiogenesis

To further determine the effect of dex on both cell types in the co-culture, immunofluorescence analyzes were performed for MyHC or CD31 after treatment with 10 and 100 µM dex. Both concentrations of dex did not influence the MyHC protein expression and did not inhibit the myotube formation in the established co-culture model. However, the formation of bulges, the building of a meshed network as well as angiogenesis and CD31 protein expression was decreased in HUVECs after the incubation with dex.

The results show that dex has different effects on different cell types. Dex inhibits angiogenesis by suppressing CD31 in mono- as well as in a co-culture with human primary myoblasts, whereas the expression of myogenic markers is not influenced by dex. Our hypothesis is that dex influences angiogenesis and thereby indirectly muscle regeneration or the development of muscle atrophy after dex treatment. One possible mechanism could be the decreased VEGF release in the co-culture after dex treatment (Figure 9), resulting in an inhibition of angiogenesis and of the formation of bulge like structures from HUVECs.

These results are confirmed by gene expression analyses: dex reduced *CD31* and *VEGF* gene expression, whereas the myogenic transcription factors *MyoD* and *MyoG* were expressed significantly higher after dex treatment, indicating that dex simultaneously had suppressing effects on endothelial cells and enhancing effects on myoblast gene expression. These enhancing effects of dex on myogenic expression were also observed in our previous study in the mono-culture of primary human myoblasts [33]. A significant downregulation of *HiF1α* gene expression was detected with both dex concentrations, indicating that dex had the potential to repress its expression and function in the co-culture and as a consequence inhibiting VEGF expression. These results show that dex has a major affinity to endothelial cells and thus influences gene expression and as a consequence, a function of these cells.

mRNA expression of GR is not significantly downregulated by dex in the co-culture but in mono-culture of HUVECs it is downregulated after treatment with 10 µM dex. This speaks to a different regulation of GR by dex in different cells, which has also been described in mouse or rat myotubes [59]. Others described that glucocorticoid treatment did not result in reduced GR mRNA and protein levels, but showed that upon dex treatment, GR is activated and translocates to the nucleus in C2C12 cells [60,61]. The protein expression analyses of CD31 (Figure 8) indicated repressing properties of dex on its expression and could function as a negative response element of the GR (nRE) under normal conditions. The two cell types stimulated each other to promote angiogenesis or myogenesis, showing that these two biological processes are coupled, which has also been described in another context [62].

In this study, we are the first to perform co-culture experiments to demonstrate cellular interactions of human endothelial cells and primary myoblasts under dex influence. This model represents an in vitro model for muscle regeneration and/or development of muscle atrophy, and demonstrates different effects of dex on different cells cultured in mono- or co-culture models.

## 4. Materials and Methods

### 4.1. Reagents

Endothelial cell basal medium (EBM-2), EGM™-2 BulletKit™ were purchased from Lonza (Wokingham, UK); EGM™-2 BulletKit™: ascorbic acid, hydrocortisone, heparin, gentamicin/amphotericin B, VEGF, epidermal growth factor (EGF), insulin-like growth factor (IGF), fibroblast growth factor (FGF), Dulbecco’s modified Eagle medium (DMEM/F-12) (1:1) + GlutaMAX and Penicillin Streptomycin, 10,000 U/mL Penicillin; 10,000 μg/mL Streptomycin were purchased from Gibco^®^ Life Technologies(Grand Island, NE, USA). Fetal Calf (FCS) and Horse serum (HS) were purchased from Biochrom GmbH (Berlin, Germany) and bFGF from BPS Bioscience (San Diego, CA, USA). RU-486 (Mifepristone) was purchased from MyBioSource (San Diego, CA, USA).

### 4.2. Cell Culture

HUVECs (primary human umbilical vein cells), purchased from PromoCell (Heidelberg, Germany) were maintained in endothelial cell basal medium (EBM-2) and the provided supplements of the EGM™-2 BulletKit™ (Table 1, HM1) at 37 °C in a humidified atmosphere containing 5% CO_2_. The medium was changed every second day. The isolation as well as characterization and differentiation of primary human myoblasts were previously described and published [33]. Muscle tissue was cut into small pieces followed by collagenase type II (470 U/DMEM-F-12, Worthington Biochemical Corporation, Lakewood, CO, USA) and Trypsin/EDTA (0.025%/0.02%, Biochrom GmbH, Berlin, Germany) treatment at 37 °C in a water bath. Cell suspensions were seeded in collagen coated culture flasks and incubated at 37 °C and 5% CO_2_. The medium (MM1) was changed every second day. Cell differentiation was induced by serum reduction with differentiation medium (DM). Cell media were supplemented with antibiotics (1% Pen.Strep). The use of residual material was approved by the ethics committee of the Landesärztekammer Rheinland-Pfalz in accordance with the principles expressed in the Declaration of Helsinki and the ICH Guidelines for GCP. All patients provided written consent.

Myoblast purity was about 80% after isolation, which was proved by FACS analyses for non-muscle cells (e.g., CD45) and immunofluorescence stainings for myoblasts (Myf5, MyoD, NCAM, MyHC) and fibroblasts (fibroblast surface protein, α-SMA). We used the C2C12 cell line as control cells for the immunofluorescence analyses (DSMZ, Braunschweig, Germany). We also proved that isolated cells were able to differentiate into myotubes confirming the myoblastic phenotype of our isolated cells [33,63,64,65].

### 4.3. Cell Viability and Proliferation Assay

To evaluate the viability and proliferation of endothelial cells after dex treatment, the alamarBlue^®^ assay was performed (Gibco^®^ Invitrogen™ Life Technologies, Carlsbad, CA, USA). Cells (1.0 × 10^4^) were seeded in a 24-well plate and incubated in HM1. After 24 h medium was refreshed and cells were incubated with or without dex for 24, 48, and 72 h. Control cells were incubated without dex. Measurements were repeated three times (*n* = 3) and analyzed using the GloMax^®^Multidetection System (Promega, Madison, WI, USA) (Ex: 525 nm; Em: 580–649 nm).

In all experiments, we used dex concentrations of 0, 10, and 100 µM as described in similar studies with murine C2C12 cells [44,45]. To show the specificity of dex, viability experiments were performed with the inhibitor RU-496 (10 µM).

### 4.4. Tube-Like Structure (TLS) Assay

HUVECs (5.0 × 10^4^ cells/well) were re-suspended in Matrigel (BD Biosciences, New Jersey, NJ, USA), seeded in a 96-well plate and incubated at 37 °C for 30 mins to allow polymerization of the gel. After that, different media compositions with 10 and 100 µM dex were added to cells for further incubation. The TLS were photographed from the same position at the center of each well after 24 h and analyzed using the Angiogenesis analyzer software in ImageJ. Images used as example to visualize this assay (not the original) were added in the Supplementary Materials.

### 4.5. Evaluation of a Co-Culture System of Primary Human Myoblasts and Endothelial Cells

**Figure 11:**
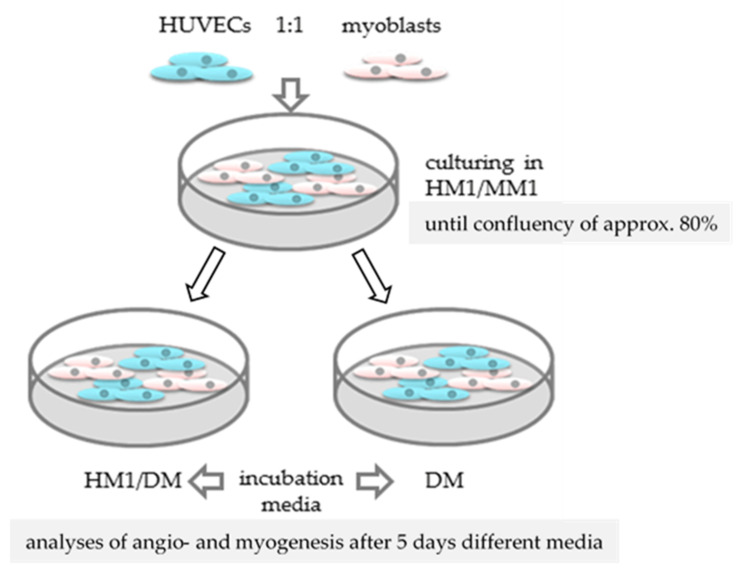
Experimental design of the co-culture evaluation with primary human myoblasts and HUVECs.

Both cell types were seeded in collagen coated tissue culture plates at the same ratio (1:1) and incubated until they reached 80% confluence (Figure 11). The co-culture growth medium (HM1/MM1) contained a 1:1 mixture of both growth media (Table 2). For induction of angiogenesis or myogenesis and to test cell behavior and their cell interactions as a co-culture, two different media compositions were used: first, the HM1/DM of HUVECs growth medium (HM1) and the differentiation medium of myoblasts (DM); and as a second, only the differentiation medium (DM) of myoblasts. Cells were incubated with both media for 5 days and analyzed.

### 4.6. Lentiviral Transduction

To allow analysis by fluorescent microscopy, cells were transduced with a lentiviral vector encoding the mCherry protein. Vector supernatants were collected and concentrated from transfected 293T producer cells as previously described [66]. For lentiviral transduction, 2 × 10^4^ HUVECs were seeded in 24-well plates. After 24 h, the medium was replaced by medium containing 5 µg/mL protaminsulfate (Sigma–Aldrich^®^GmbH, St. Louis, MO, USA). Subsequently, 20 µL of a 1:10 in medium diluted virus suspension was pipetted into each well and incubated for 6 h. Flow cytometry analyses one week after transduction confirmed a transduction efficiency of 90%.

### 4.7. Immunofluorescence

To detect angiogenesis or myogenesis in the co-culture, cells were stained with the specific antibody MyHC for myotubes (myogenesis) and CD31 for HUVECs (angiogenesis) and immunofluorescence analyses were performed.

After washes with PBS (Gibco^®^Invitrogen™ Life Technologies, Carlsbad, CA, USA), cells were fixed and permeabilized with methanol for 20 min followed again by washes with PBS. The following primary antibodies were used and incubated over night at 4 °C. Skeletal Muscle MyHC (F59: sc-32732, 1:200, Santa Cruz Biotechnology, Dallas, TX, USA) and CD31 (ab28364, 1:50, Abcam, Cambridge, GB). After washes with BSA/PBS cells were stained with the secondary antibody Alexa Fluor^®^488 (A11001 and A11008, 1:200, Invitrogen™ Life Technologies, Carlsbad, CA, USA) for 1 h in the dark at room temperature. Nuclei staining was performed with Hoechst dye (Hoechst dye 334565, Sigma–Aldrich^®^GmbH, St. Louis, MO, USA) before detection using the EVOS^®^ Digital Inverted Microscope (EVOS fl, Life Technologies, Carlsbad, CA, USA). Experiments were performed three times.

### 4.8. Quantitative Real-Time Polymerase Chain Reaction (qPCR)

Total RNA isolation from HUVECs in Matrigel was performed using TRIzol reagent (Invitrogen™, Life Technologies, Paisley, UK). RNA from all other cell experiments was isolated with the RNA isolation kit (peqGold, total RNA kit, PEQLAB Biotechnology GmbH, Erlangen, Germany) according to manufacturer instructions followed by quantification using UV spectroscopy.

1 µg of total RNA was reverse transcribed into cDNA using dNTPs (4you4 dNTPs mix (10 mM), BIORON GmbH, Ludwigshafen, Germany), random primers (Promega, Madison, WI, USA) and MuLV RT (M-MuLV Reverse Transcriptase, M0253S New England Biolabs, Ipswich, USA) according to manufacter instructions.

For gene expression analysis, cDNA template underwent PCR amplification (40 cycles) using the Biozym Blue S´Green Master Mix (Biozym Scientific GmbH, Hessisch Oldendorf, Germany) and sequence specific primers (Primer sequences are listed in Table 3) for human *CD31*, *VEGF*, *IL-6*, -*10*, *HiF1α*, *MyoD*, and *MyoG*. *GAPDH* was used to normalize gene expression. Sample amplification was performed with the qTower3 (Jena Analytik, Jena, Germany). An initial denaturation step at 95 °C for 2 min, denaturation, and enzyme activation at 95 °C for 5 sec followed by 60 °C for 30 sec for annealing and extension were performed. Results were calculated using the 2^−ΔΔ^Ct method [55]; they presented the expression levels of cells relative to gene expression of untreated cells.

### 4.9. Enzyme-Linked Immunosorbent Assay (ELISA)

To test if our primary myoblasts secreted any angiogenesis induced growth factors, VEGF ELISA was performed using the human VEGF, DuoSet^®^ (Elisa Development System, R&D systems™ Inc., Minneapolis, MN, USA) from the co-culture without any added growth factors 24, 48, 72, and 96 h after switching the medium from growth to differentiation medium (DM). Experiments were repeated three times and measurements were performed in duplicates. Absorbance was measured at 450 nm using a Dynex microplate reader (DYNEX TECHNOLOGIES, Buštěhrad, Czech Republic).

### 4.10. Statistical Analysis

Statistical analyses were performed using SPSS (IBM^®^ GmbH, Ehningen, Germany). The results are presented as medians and quartiles or as mean ± standard deviation. Measurements were carried out in duplicates or triplicates. Experiments were independently repeated three times. Normally distributed data were analyzed by one-way ANOVA and pairwise comparisons were conducted post hoc test. Non-normally distributed data were evaluated with the Kruskal–Wallis test. For pairwise comparisons, the Mann–Whitney-U test was used and *p*-values < 0.05 were considered statistically significant (* *p* < 0.05).

## 5. Conclusions

In the present study, we investigated the effect of the GC dex on endothelial cells in a mono-culture and in a co-culture with myoblasts under different growth conditions. To our knowledge, we are the first to establish and evaluate a co-culture of primary human myoblasts and HUVECs, to analyze their growth behavior and the impact of dex on both cell types with regard to development of muscle atrophy.

We found that dex had different effects on different cell types in mono- and in a co-culture system. We observed a cell type specific affinity of dex to HUVECs and the repression of angiogenic genes and proteins, and as a result repressed angiogenesis in a co-culture. Furthermore, dex impaired VEGF release of myoblasts and as a consequence, angiogenesis. These results indicate that dex affects angiogenesis via inhibition of VEGF release at least in myoblasts, which could be responsible not only for the development of muscle atrophy after dex administration, but also for inhibition muscle regeneration in combination with vascular damage.

## Figures and Tables

**Table 1:** Cell culture media and different HUVEC cell media and kits.

Cell Type	Medium	Formulation
HUVEC	growth medium (HM1)	5% FCS, EBM-2, EGM™-2 BulletKit™
HUVEC	growth medium without VEGF (HM1-V)	5% FCS, EBM-2, EGM™-2 BulletKit™, except VEGF
HUVEC	without supplementation (EBM-2)	EBM-2
HUVEC	without growth factors (GF) (EBM-2 Ø GF)	EBM-2 + 5% FCS, ascorbic acid, heparin, gentamicin/amphotericin B
Myoblasts	growth medium (MM1)	DMEM/F-12, 10% FCS, 2.5 ng/mL bFGF
Myoblasts	differentiation medium (DM)	DMEM/F-12, 5% HS
CoCulture	induction medium for myo-or angiogenesis medium (1:2) HM1/DM	EBM-2 + EGM-2 Bullet Kit DMEM/F-12, 5% HS (1:2)

**Table 2:** Media composition for co-culture evaluation.

growth medium HM1/MM1 (1:2)	EBM-2 + EGM™-2 BulletKit™, DMEM/F-12, 10% FCS, 2.5 ng/mL bFGF
1:2 medium HM1/DM (1:2)	EBM-2 + EGM™-2 BulletKit™, DMEM/F-12, 5% HS
differentiation medium (DM)	DMEM/F-12, 5% HS

**Table 3:** Primer sequences for qPCR analysis (Eurofins Genomics, Ebersberg, Germany).

Primer	Sequence
*GAPDH* Acc.# M33197	FW: cgaccactttgtcaagctca
	RV: aggggagattcagtgtggtg
*CD31* Acc.# NM_000442	FW: cattggcgtgttgggaagaa
	RV: gctcatgtttgcctagctcc
*VEGF* Acc.# M32977	FW: agatgagcttcctacagcacaac
	RV: aggacttataccgggatttcttg
*IL-6* Acc.# NM_000600	FW: cacagacagccactcacctc
	RV: cctcaaactccaaaagacca
*IL-10* Acc.# NM_000572	FW: cgtggagcaggtgaagaatg
	RV: atagaaatgggggttgaggt
*HiF1α* Acc.# NM_001243084	FW: gaaaacttggcaaccttgga
	RV: atctccgtccctcaacctct
*GR* Acc.# AB307716	FW: caaatcagcctttcctcggg
	RV: ctggcccttcaaatgttgct
*MyoD* Acc.# X56677.1	FW: ggggctaggttcagctttct
	RV: gctctggcaaagcaactctt
*MyoG* Acc.# NM_002479.5	FW: gccagactatccccttcctc
	RV: gaggccgcgttatgataaaa

## Data Availability

The datasets used and/or analysed during the current study are available from the corresponding author on reasonable request.

## Supplementary Materials

The following are available online at https://www.mdpi.com/article/10.3390/ijms22157986/s1.
