# Supplementary material for: Dexamethasone Inhibits the Pro-Angiogenic Potential of Primary Human Myoblasts
Source: Int J Mol Sci. 2021 Jul 26;22(15):7986. doi: 10.3390/ijms22157986 (PMC8348204; doi:10.3390/ijms22157986)
Supplement: Supplementary file 1 [file ijms-22-07986-s001.zip › ijms-1289034-supplementary.pdf]

## Supplementary Material

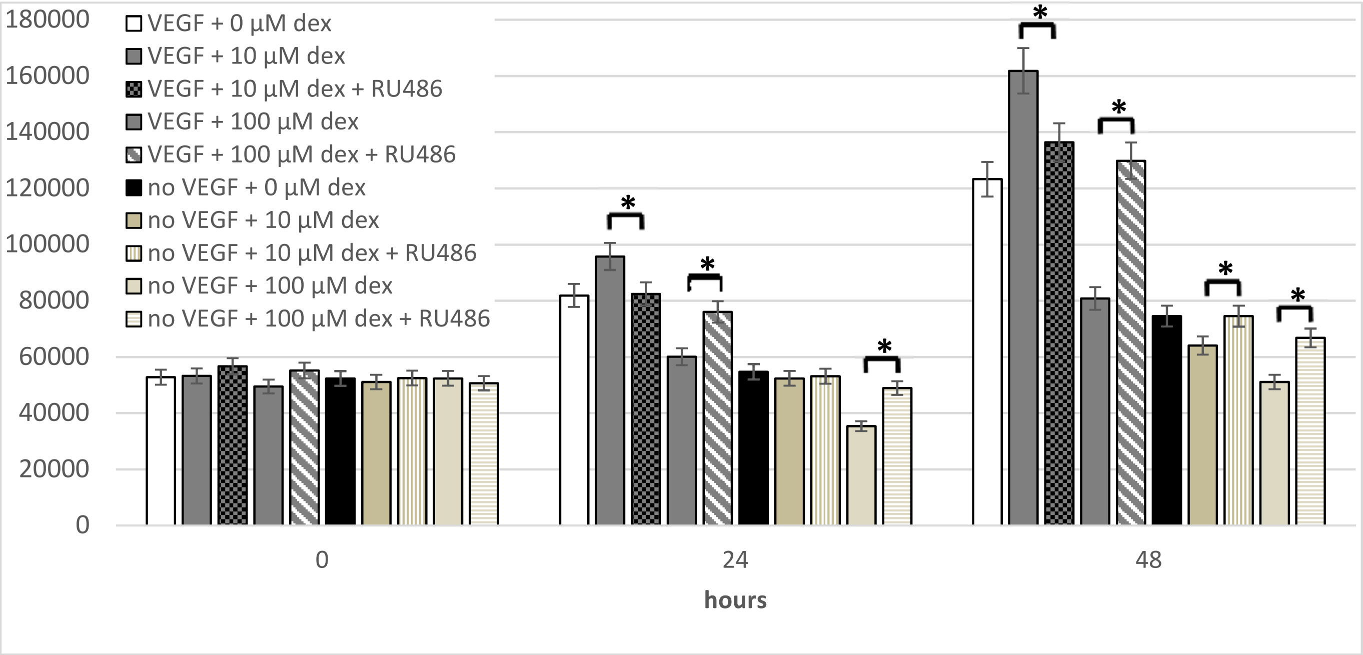


**Figure S1.**. Viability assay of HUVECs after 0, 24, and 48 h in HM1 medium (with VEGF) or in HM1-V medium (without VEGF) and dex (10, 100 µM) and GR antagonist RU-486 (10µM). Results are presented as bar charts, error bars show the standard deviation (n = 3). A *p* value < 0.05 indicates statistical significance (* $p < 0.05$), n.s. = non-significant. Significant changes are only indicated for dex with and without GR antagonist, to illustrate the reverse effects after addition of inhibitor.

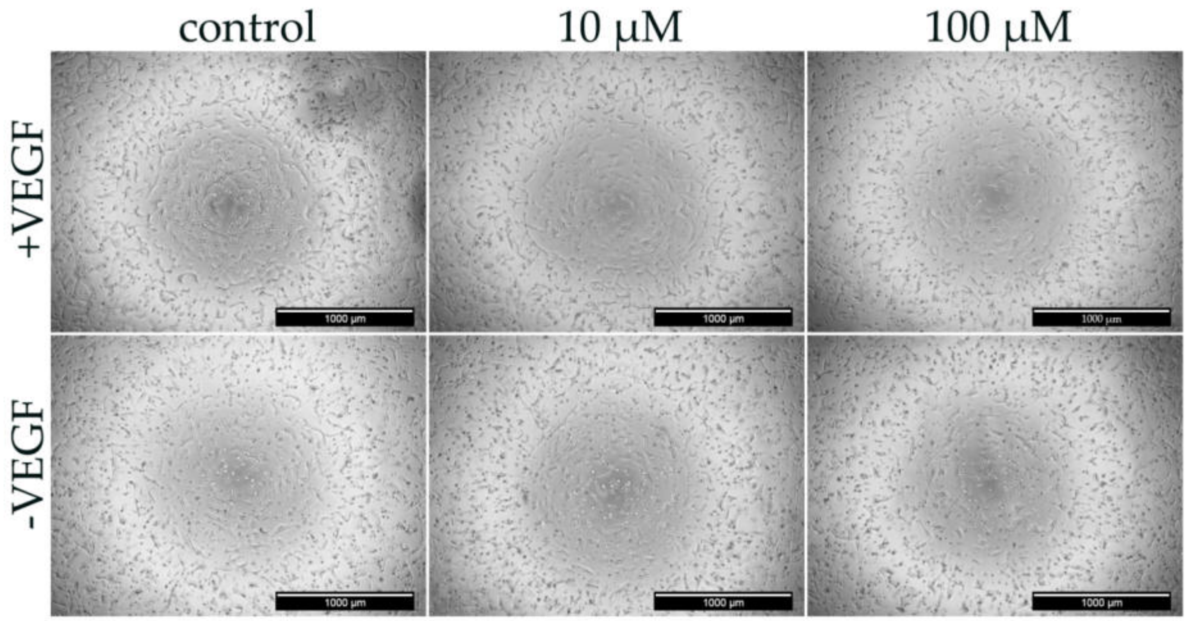


**Figure S2**. Representative images of the TLS after 24 h. HUVECs were maintained in HM1 with VEGF or in HMV-1 without VEGF and incubated with 10 µM and 100 µM dex.

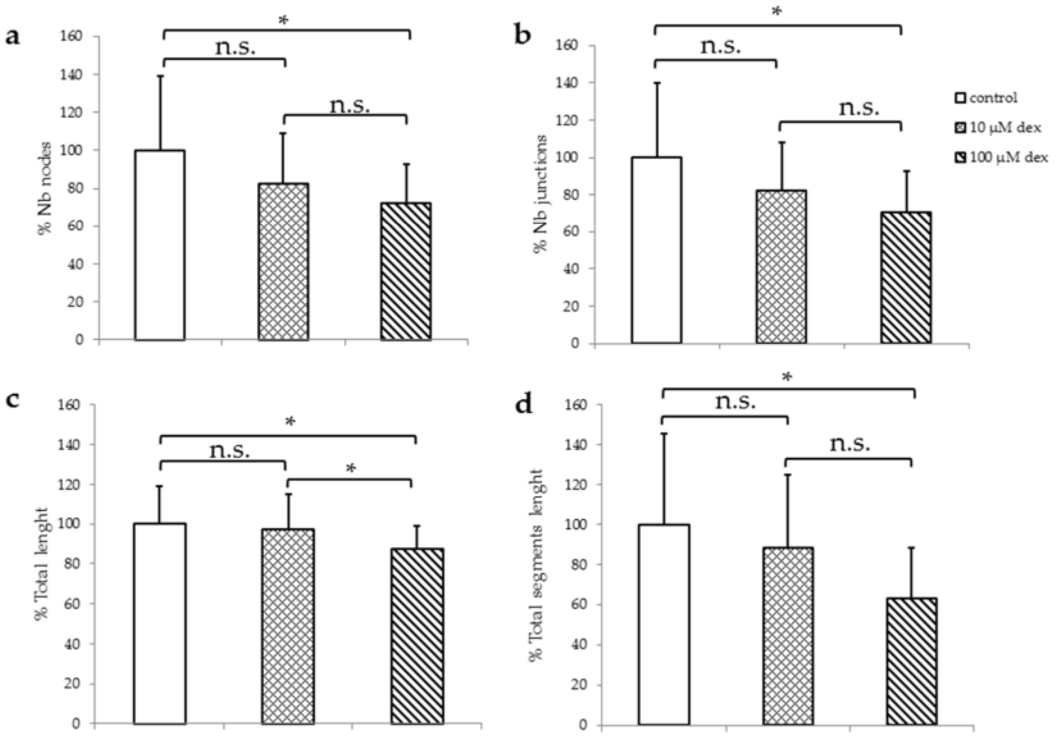


**Figure S3**. TLS assay of HUVECs after 24 h in HMV-1 (medium without VEGF) with 10 and 100 μM dex. Results are presented as bar charts, error bars show the standard deviation (n = 3). Quantitative analyses were performed for number of nodes (a), number of junctions (b), total length (c), and the total segments length (d). Data were normalized to a control of 100%. A $p$ value < 0.05 indicates statistical significance (* $p < 0.05$), n.s. = non-significant.

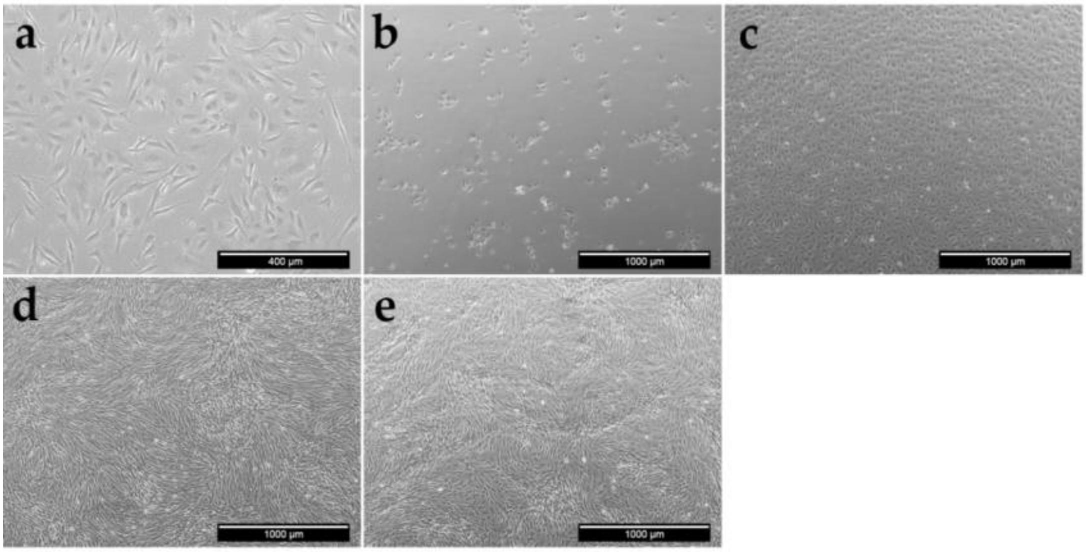


**Figure S4.** Representative images of the co-culture evaluation. Cells are shown in mono as well as in co-culture using different media compositions. a: Co-culture in HM1/MM1, (1:2), b: HUVECs in DM, c: HUVECs in HM1/DM, (1:2), d: Co-culture in HM1/DM, (1:2), e: Co-culture in DM.

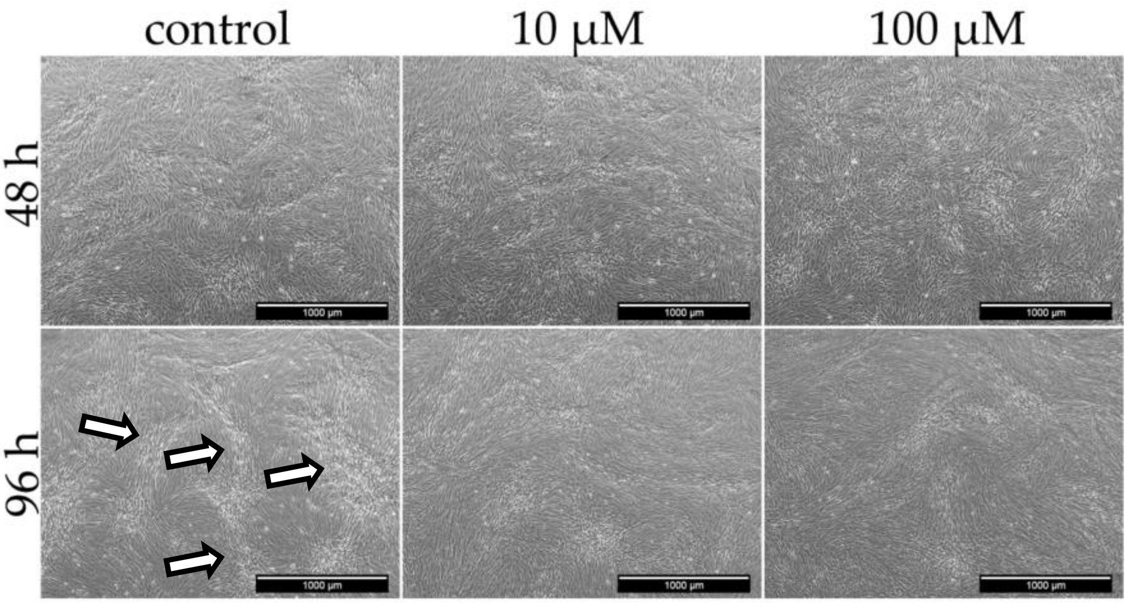


**Figure S5.** Representative images of the co-culture after 48 and 96 h incubation with 10 and 100 μM dex.
